# Supplementary material for: Epidemic-induced local awareness behavior inferred from surveys and genetic sequence data
Source: Nat Commun. 2025 May 22;16:4758. doi: 10.1038/s41467-025-59508-5 (PMC12098690; doi:10.1038/s41467-025-59508-5)
Supplement: Supplementary file 1 — Supplementary Information [file 41467_2025_59508_MOESM1_ESM.pdf]

Supplementary Material for Epidemic-induced local awareness behavior inferred from surveys and genetic sequence data

A Detailed explanation of superspreading event detection and ECS assignment

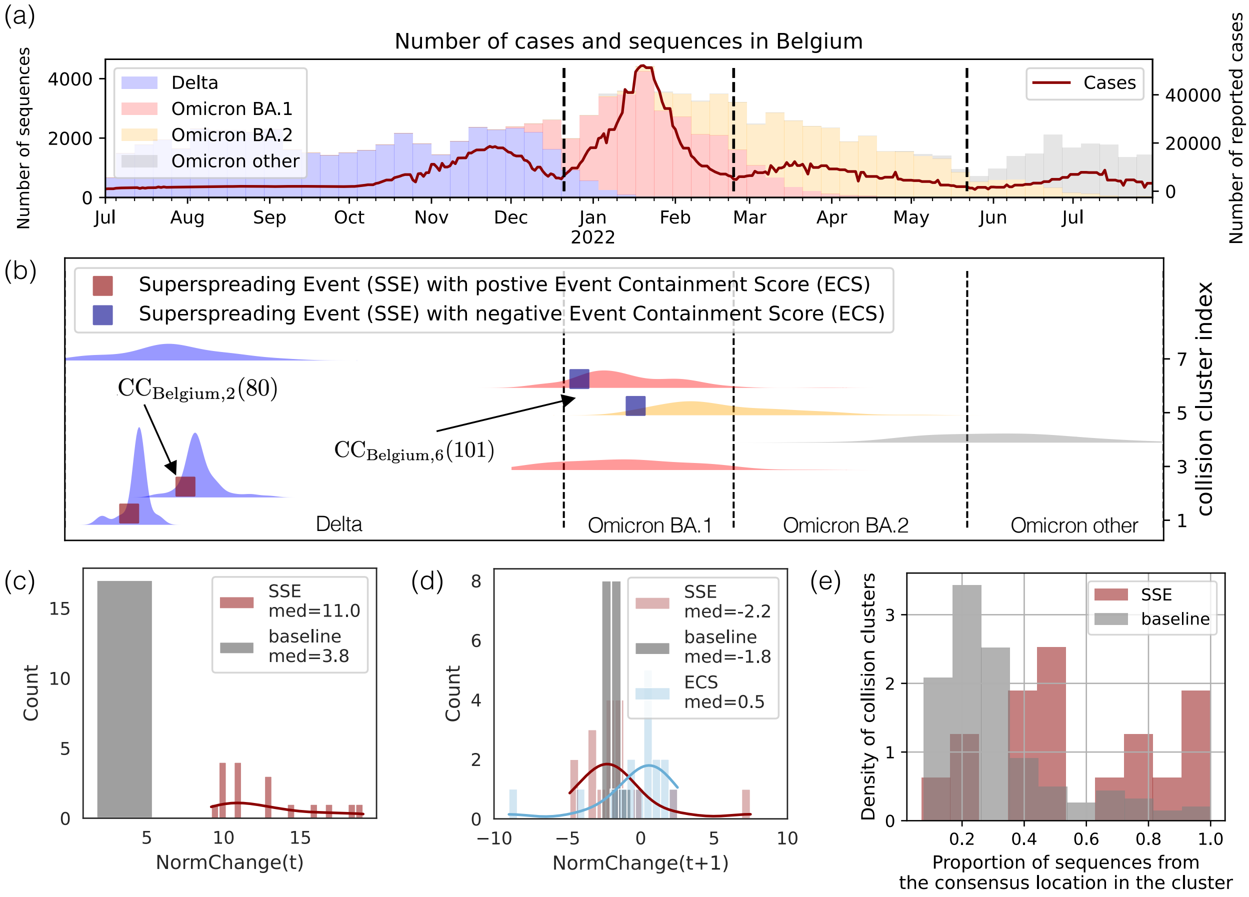

Figure A.1: (a) Bar plot showing the number of SARS-CoV-2 genetic sequences collected in Belgium and uploaded to the GISAID platform over time, for the Delta and the early Omicron variants. (b) Visualization of the sizes of 7 collision clusters in Belgium over time. Each individual plot shows the number of sequences in the collision cluster at a given date (denoted by $\mathrm{CC}_{c,i}(t)$). The red squares (located often towards the beginning of a collision cluster) mark the superspreading events detected using our proposed method. (c) Histogram of the $\mathrm{NormChange}_{c,i}(t)$ and the $\mathrm{Baseline}_{c,i}(t)$ values in Belgium during the Delta wave. By definition, these values are larger than 9 for superspreading events (SSEs), and at most 9 for baselines. (d) Histogram of the $\mathrm{NormChange}_{c,i}(t+1)$, the $\mathrm{Baseline}_{c,i}(t+1)$ values, and the resulting ECS values in Belgium during the Delta wave. The outcome of the pipeline – the $\mathrm{ECS}_{\mathrm{Belgium,Delta}}$ value – is the median of the plotted ECS values (in this case 0.5). (e) The histogram of the proportion of sequences that belong to the most frequently appearing (consensus) location in each detected superspreading and baseline event shows that superspreading events are more spatially localized.

We index collision clusters only by the time t (integer value measured in weeks since the first sequence), their country-variant pair denoted by c, and their cluster index i (Figure A.1 (b)). In order to track changes in collision cluster sizes, we are interested in the Normalized Change values defined as

$$\mathrm{NormChange}_{c,i}(t) = \frac{\mathrm{CC}_{c,i}(t+1) - \mathrm{CC}_{c,i}(t)}{\max(1, \sqrt{\mathrm{CC}_{c,i}(t)})}, \tag{1}$$

where $\mathrm{CC}_{c,i}(t)$ denotes the size of the collision cluster indexed by (c, i, t). The normalization with the square root of the collision cluster size accounts for the natural fluctuation of the cluster sizes. Indeed, assuming that the patients in the collision clusters at time t independently infect an identically distributed random number of new patients with the same amino acid signature at time $t+1$, by the Central Limit Theorem, we expect the fluctuations of $\mathrm{CC}_{c,i}(t+1)$ to be proportional to the square root of $\mathrm{CC}_{c,i}(t)$. Due to this normalization, NormChange values tend to be close to zero; in most countries 95% of the values fall between -3 and 5. We consider exceptionally large NormChange values as a sign of a superspreading event. Inspired by [1], we choose the threshold for the NormChange value of a superspreading event to be 9, and we provide a robustness analysis on this threshold parameter in Section B.1. The proposed superspreading event detection method is efficient, requires only minor preprocessing, and the detected superspreading events agree with our intuition after visual inspection (Figure A.1 (b)).

Similarly to previous superspreading event detection methods based on thresholding genetic sequence counts [2, 1], our proposed method is imperfect, leading to both false positives and false negatives. However, since we only apply aggregate statistics on the identified superspreading events, even such imperfect methods can provide important results, especially if the confounding factors can be ruled out. The main confounding factor in this case is sampling bias, as we know that different countries collected and sequenced samples with different strategies and at different rates [3]. To control for country-specific biases, we match each superspreading event (c, i, t) with multiple baseline collision cluster timesteps with the same country-variant index c and with similar sampling time.

We denote the median of the NormChange(t) values of the baselines as $\mathrm{Baseline}_{c,i}(t)$ (see Methods). As shown in Figure A.1 (c), the NormChange values at t of superspreading events are all larger than a threshold and follow broad distribution, whereas the distribution of the $\mathrm{Baseline}_{c,i}(t)$ values is concentrated below the threshold . Once the baselines are matched, we define our main notion of interest, the ECS, as the difference between the baseline value and the superspreading event NormChange value at time $t+1$:

$$\mathrm{ECS}_{c,i}(t) = \mathrm{Baseline}_{c,i}(t+1) - \mathrm{NormChange}_{c,i}(t+1). \tag{2}$$

We present the distribution of $\mathrm{NormChange}_{c,i}(t+1)$ values for superspreading events, the $\mathrm{Baseline}_{c,i}(t+1)$ values for baseline events, along with the resulting ECS values in Belgium, in Figure A.1 (d). Since all country-variant pairs c in our dataset had similarly broad, but unimodal ECS distributions as Figure A.1 (d), we focused on their median values denoted by MECS. As the non-synonymous mutation rate of SARS-CoV-2 (we estimated a non-synonymous mutation probability of around 0.32 during a transmission event) was higher than the effective reproduction rate (often below 1.5), and collision clusters can be thought of as sub-critical spreading processes (with expected offspring number smaller than $(1 - 0.32) \cdot 1.5 \approx 1$), it is no surprise that the median values of the $\mathrm{NormChange}_{c,i}(t+1)$ values are negative for both the superspreading events and the baselines. However, the sign of MECS adds non-trivial information. A positive MECS means that the normalized change of the number of genetic sequences in superspreading events was smaller than in the baseline, which suggests that the superspreading events led to fewer secondary infections than a similarly sized non-superspreading clusters of infectious individuals, i.e. the superspreading events were well-contained. Similarly, a negative MECS would suggest superspreading events that were not contained as well as the baselines in the same country during the same variant.

Since in Belgium the GISAID metadata contains settlement-level location information of the collected sequences, we were able to include additional validation steps for the detected superspreading events and baselines. For each superspreading and baseline event, we compute a consensus location (the most frequent location) of the samples. For superspreading events, we expect that most samples come from the consensus location, whereas for baseline events we expect that the samples come from a mixture of locations, and only a few from the consensus location. Indeed, this is what we observe when we plot a histogram of the proportion of samples that belong to the consensus location in Figure A.1 (e). Unfortunately, such fine-grained information is not available in other countries, prohibiting a similar analysis on the entire dataset.

B Robustness analyses

B.1 Threshold for superspreading event Detection

We detect superspreading events by applying a threshold on the $\mathrm{NormChange}_{c,i}(t)$ values defined in equation (1). By default, this threshold is set to be 9 following [1], who chose this value based on the theoretical justification of [4]. A notable difference between our approach and the referenced papers is that they assume the superspreading events to start from a single source, which can be identified in the dataset (e.g. via contact tracing), and they apply the threshold on the number of secondary cases of the source. In our approach, we do not assume that we can identify the source of the superspreading event, we are only interested in detecting

the occurrence of superspreading events based on collision cluster sizes. For instance, if an $\mathrm{CC}_{c,i}(t) = 10$ and $\mathrm{CC}_{c,i}(t+1) = 100$, then we suspect that this unexpected increase is due to a superspreading event that occurred at t, but we do not know which patient caused the SSE. In principle, it is possible that not one but multiple patients with the same amino acid signature caused independent and simultaneous superspreading events, however, since this is an unlikely event, we can safely ignore it without significantly impacting our aggregate statistics. In our approach, it is important to also account for the fact that $\mathrm{CC}_{c,i}$ changing from 5 to 50 is not the same as a change from 500 to 545, as larger collision cluster sizes also have larger natural fluctuations. Assuming that (due to the Central Limit Theorem), if no superspreading event occurs, collision cluster sizes behave similarly to Gaussian random variables with their mean and variance proportional to $\mathrm{CC}_{c,i}(t)$, we normalize the collision cluster size changes by the square root of $\mathrm{CC}_{c,i}(t)$ in the definition of the NormChange function. When $\mathrm{CC}_{c,i}(t) = 1$, then we get back the setup of [1], which motivated us to choose the same threshold for superspreading event detection as they did.

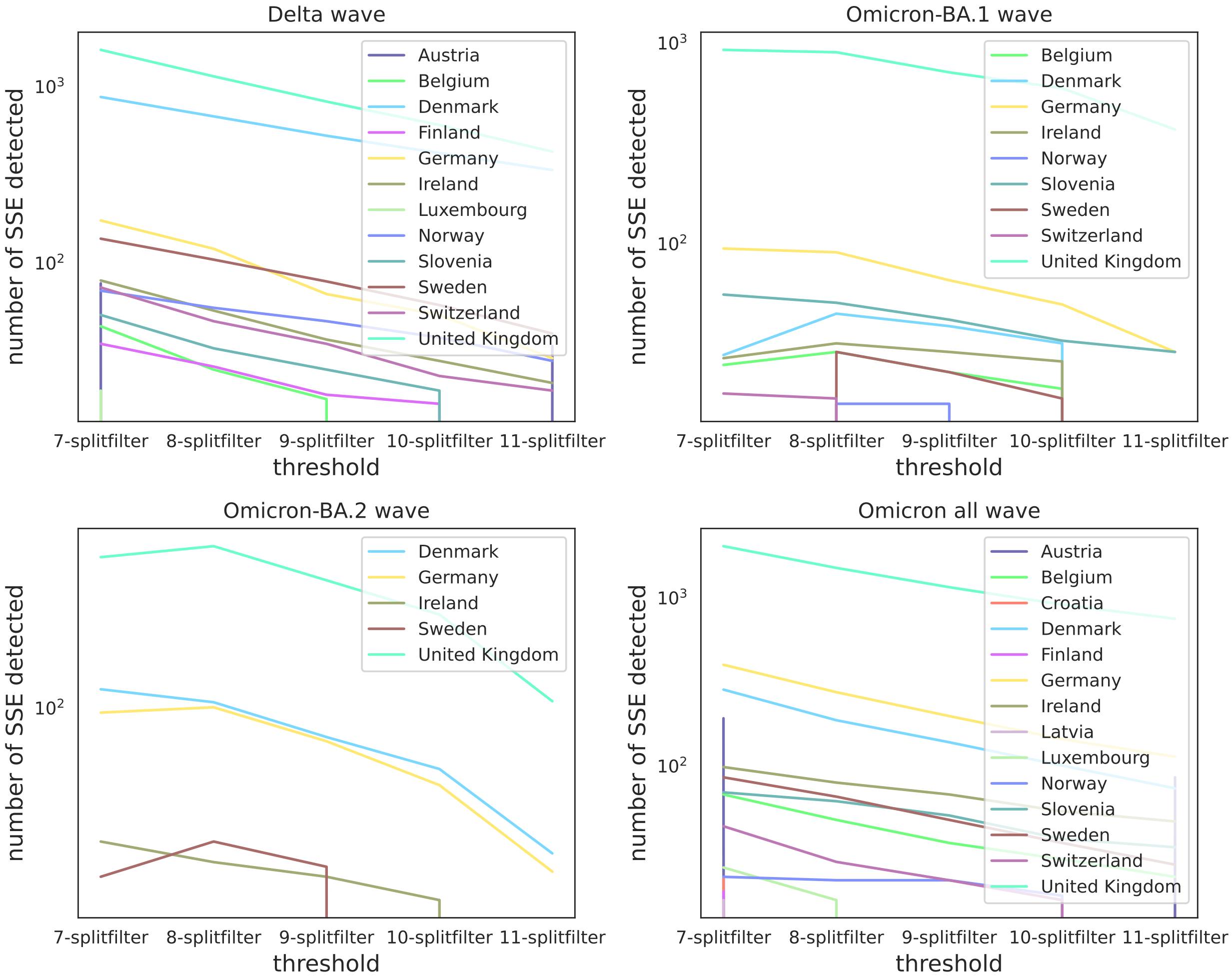

Figure B.1: The number of superspreading events detected at different thresholds stratified by country. Only datapoints with y-value above 15 are shown. As the threshold increases, fewer and fewer events are classified as superspreading events.

To further strengthen the validity of our results, we present a robustness analysis on the threshold parameter. First, in Figure B.1 we show the number of detected superspreading events in various European countries as a function of the threshold parameter, if at least 15 superspreading events were detected (and therefore qualified for our analysis). As expected, the number of superspreading events is a monotone decreasing function of the threshold. Moreover, due to the log-scale it appears that the number of detected superspreading events decrease exponentially with the threshold, indicating that it is sufficient to perform the robustness analysis in a relatively narrow parameter range. We selected the interval [7,11] because a threshold of 11 only detects a minimal number of superspreading events in many countries, making them ineligible for our analysis, while a threshold of 7 results in a high number of superspreading events, potentially leading to an excessive number of false positives. For completeness we also report the number of superspreading events corresponding to each MECS value shown in Figure 4 of the main text in Table B.1.

(a) Delta variant		(b) Omicron BA.1 variant		(c) Omicron all variants	
country	number of ST	country	number of ST	country	number of ST
Sweden	79	United Kingdom	717	Sweden	48
Switzerland	35	Sweden	23	Ireland	68
Slovenia	25	Belgium	23	United Kingdom	1163
Denmark	529	Germany	66	Denmark	139
United Kingdom	826	Slovenia	42	Belgium	35
Norway	47			Germany	199
Belgium	17			Slovenia	51
Ireland	37				
Germany	67				

Table B.1: The number of superspreading events corresponding to each MECS value in Figure 4.

In Figures B.2-B.4, we recreated Figure 4 of the main text for each integer superspreading event detection threshold in the range [8,10], for all of the major SARS-CoV-2 variants. While there is some variability in the results for different thresholds (mostly due to new countries entering the dataset as the threshold decreases), besides the correlation between the MECS and the sampling date in the Delta wave also mentioned in the main text, the most significant correlations remain between the MECS and the Containment Health Index (CHI) in the Delta and the Omicron waves. These additional results further strengthen the conclusion made in the main text, that MECS is most correlated with the CHI (the most direct measure of human behavior) among the available exogenous variables, which includes potential confounding factors (sequencing rate, attack rate).

B.2 Threshold for the Number of Baseline Events

In the Methods section, we defined a parameter m, which sets the minimum number of baseline events that are matched with each the detected superspreading event in the dataset. We expect that if we chose one baseline event, then the results could look very noisy, therefore we set $m = 5$ to ensure at least $2m = 10$ baseline events by default. In Figures B.5-B.6 we recreate Figure 4 of the main text with $m \in \{2, 5, 10\}$ to show that the precise value of m is is not important, as long as m is sufficiently high.

B.3 Simulation parameters

In the Methods section, the default epidemic model parameters were chosen so that they generally match the reported values for the COVID-19 pandemic, acknowledging the fact that many of these parameters are difficult to pin down precisely, and have likely changed over time. The default infection probability $\beta_0 = 0.15$ is chosen to be in the range of the secondary attack rate reported for households in the beginning ([4.6%, 49.56%]) [5], and and during the Omicron variant ([14.3%–17.5%]) [6], so that the epidemic is supercritical on every model network. Due to the uncertainty regarding this parameter, we perform a parameter sweep on β_0 in Figure 3 (c) of the main text. The default recovery rate $\gamma = 0.3$ is selected so that individuals are expected to recover after by the third week of the infection, agreeing with the general estimates published for SARS-CoV-2 [7]. We do not explore the sensitivity of the model to this parameter, since in most cases it is the ratio β_0/γ that determines the behavior of the epidemic model (up to time scaling). As discussed in the Methods section, we set the mutation probability at a single site to be $p_{mut} = 0.0375$ to match estimates on the non-synonymous mutation probability of SARS-CoV-2 during a transmission event.

References

[1] J. E. Lemieux, K. J. Siddle, B. M. Shaw, C. Loreth, S. F. Schaffner, A. Gladden-Young, G. Adams, T. Fink, C. H. Tomkins-Tinch, L. A. Krasilnikova, *et al.*, "Phylogenetic analysis of sars-cov-2 in boston highlights the impact of superspreading events," *Science*, vol. 371, no. 6529, p. eabe3261, 2021.

[2] X. Bello, J. Pardo-Seco, A. Gómez-Carballa, H. Weissensteiner, F. Martinón-Torres, and A. Salas, "Covidphy: A tool for phylogeographic analysis of SARS-CoV-2 variation," *Environmental Research*, vol. 204, p. 111909, 2022.

[3] T. R. Mercer and M. Salit, "Testing at scale during the COVID-19 pandemic," *Nature Reviews Genetics*, vol. 22, no. 7, pp. 415–426, 2021.

[4] J. O. Lloyd-Smith, S. J. Schreiber, P. E. Kopp, and W. M. Getz, "Superspreading and the effect of individual variation on disease emergence," *Nature*, vol. 438, no. 7066, pp. 355–359, 2005.

[5] K. Shah, D. Saxena, and D. Mavalankar, "Secondary attack rate of COVID-19 in household contacts: a systematic review," *QJM: An International Journal of Medicine*, vol. 113, no. 12, pp. 841–850, 2020.

[6] P. H. England, "SARS-CoV-2 variants of concern and variants under investigation in england," *Technical briefing*, vol. 23, 2021.

[7] D. Baud, X. Qi, K. Nielsen-Saines, D. Musso, L. Pomar, and G. Favre, "Real estimates of mortality following COVID-19 infection," *The Lancet infectious diseases*, vol. 20, no. 7, p. 773, 2020.

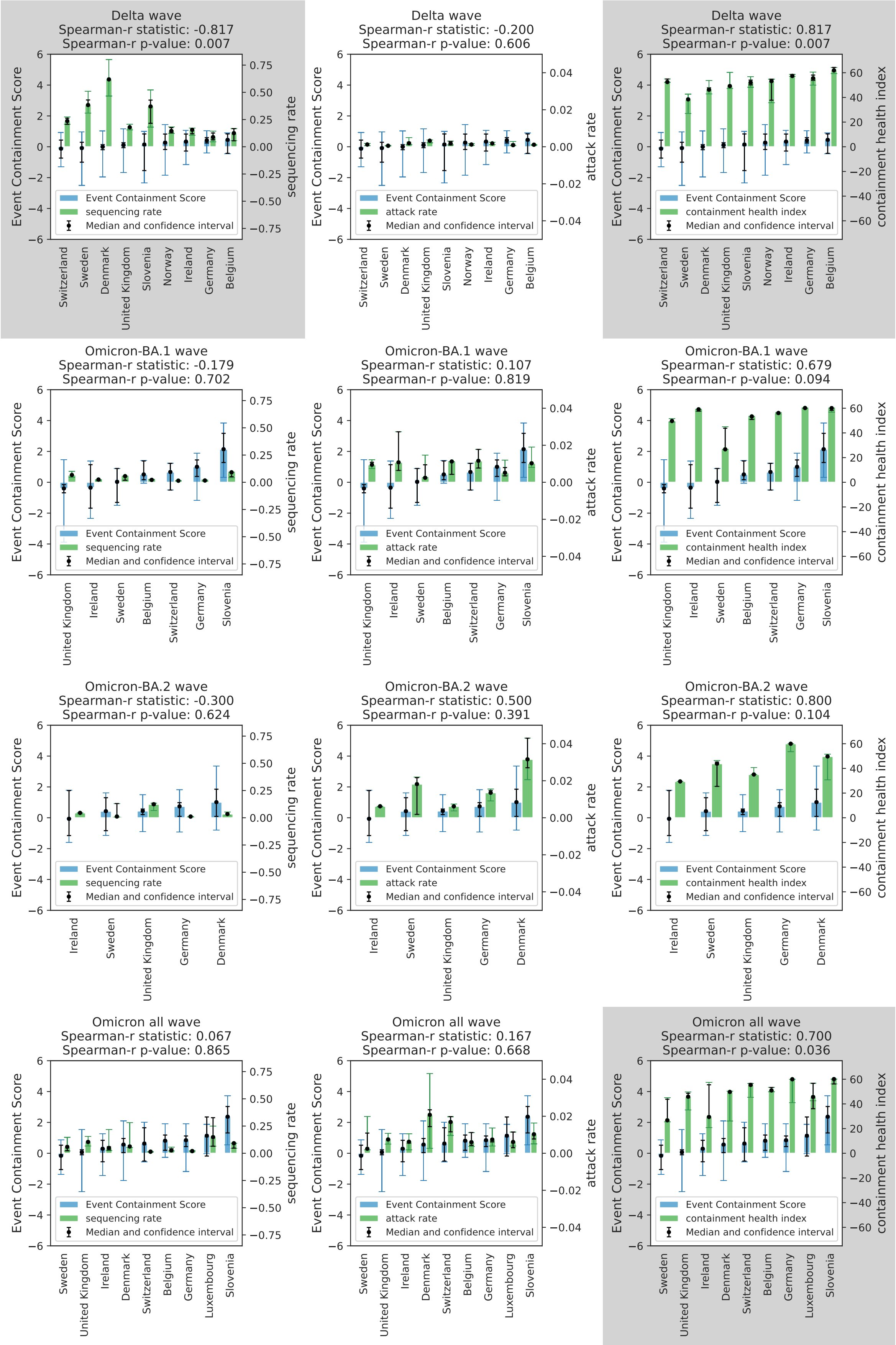

Figure B.2: The figure shows how Figure 4 of the main text would look like if a threshold of 8 was chosen instead of the default value (9) for the Delta and Omicron waves. Grey background signifies a statistically significant correlation between the MECS and the exogenous variable. Error bar definitions and all plotting parameters are the same as in Figure 4.

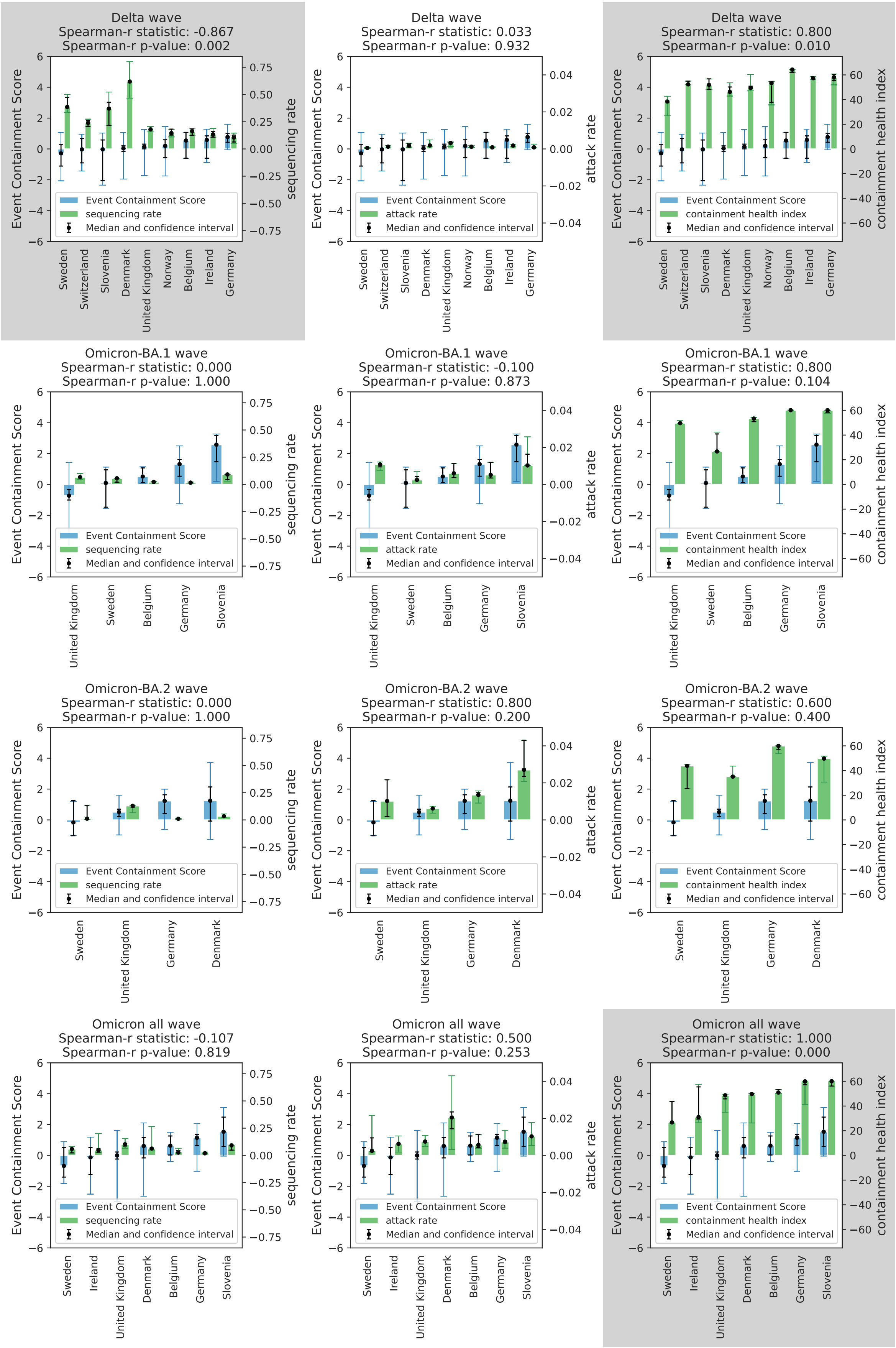

Figure B.3: The figure shows how Figure 4 of the main text for the Delta and Omicron waves with the default threshold (9). Grey background signifies a statistically significant correlation between the MECS and the exogenous variable. Error bar definitions and all plotting parameters are the same as in Figure 4.

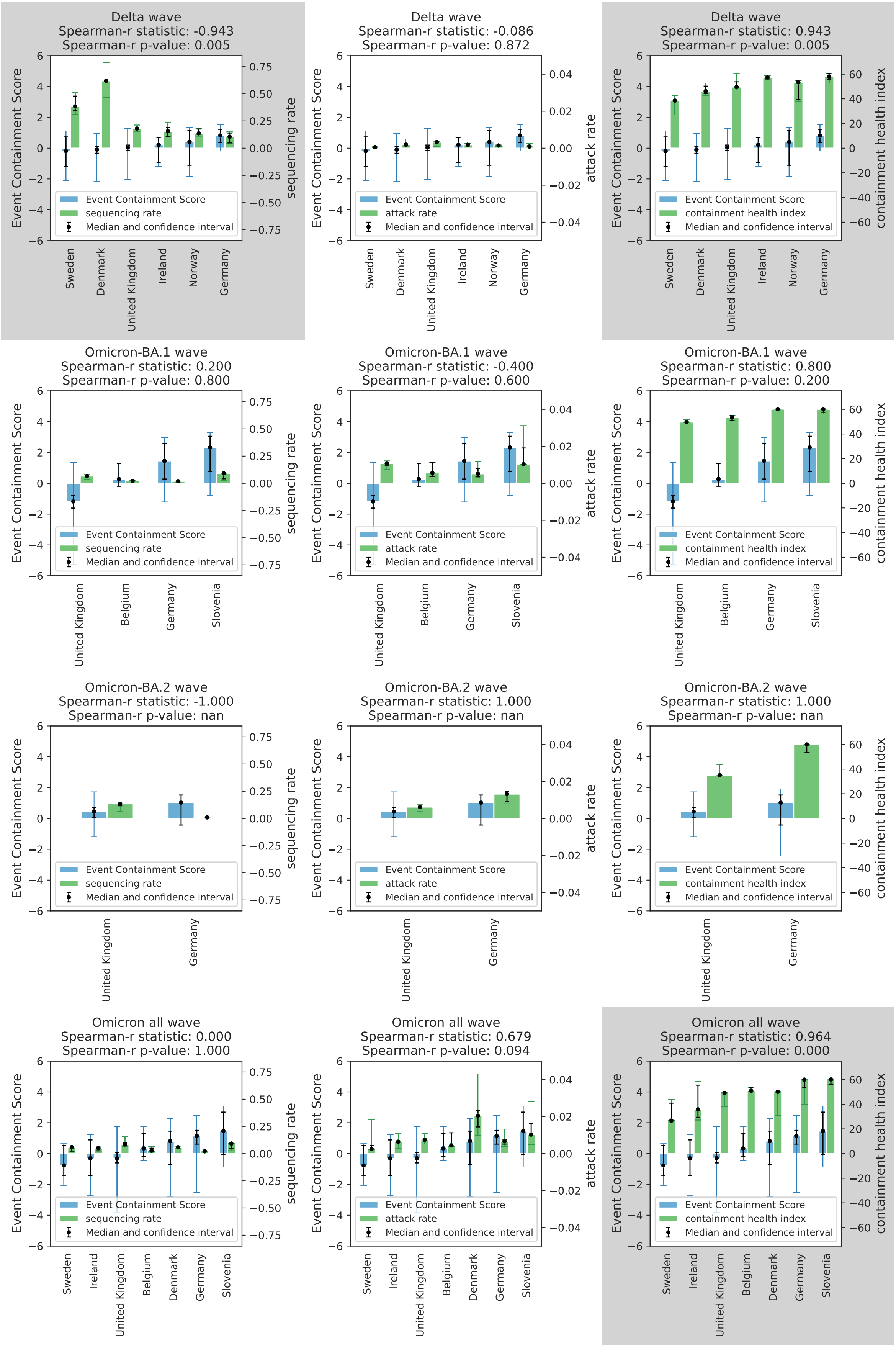

Figure B.4: The figure shows how Figure 4 of the main text would look like if a threshold of 10 was chosen instead of the default value (9) for the Delta and Omicron waves. Grey background signifies a statistically significant correlation between the MECS and the exogenous variable. Error bar definitions and all plotting parameters are the same as in Figure 4.

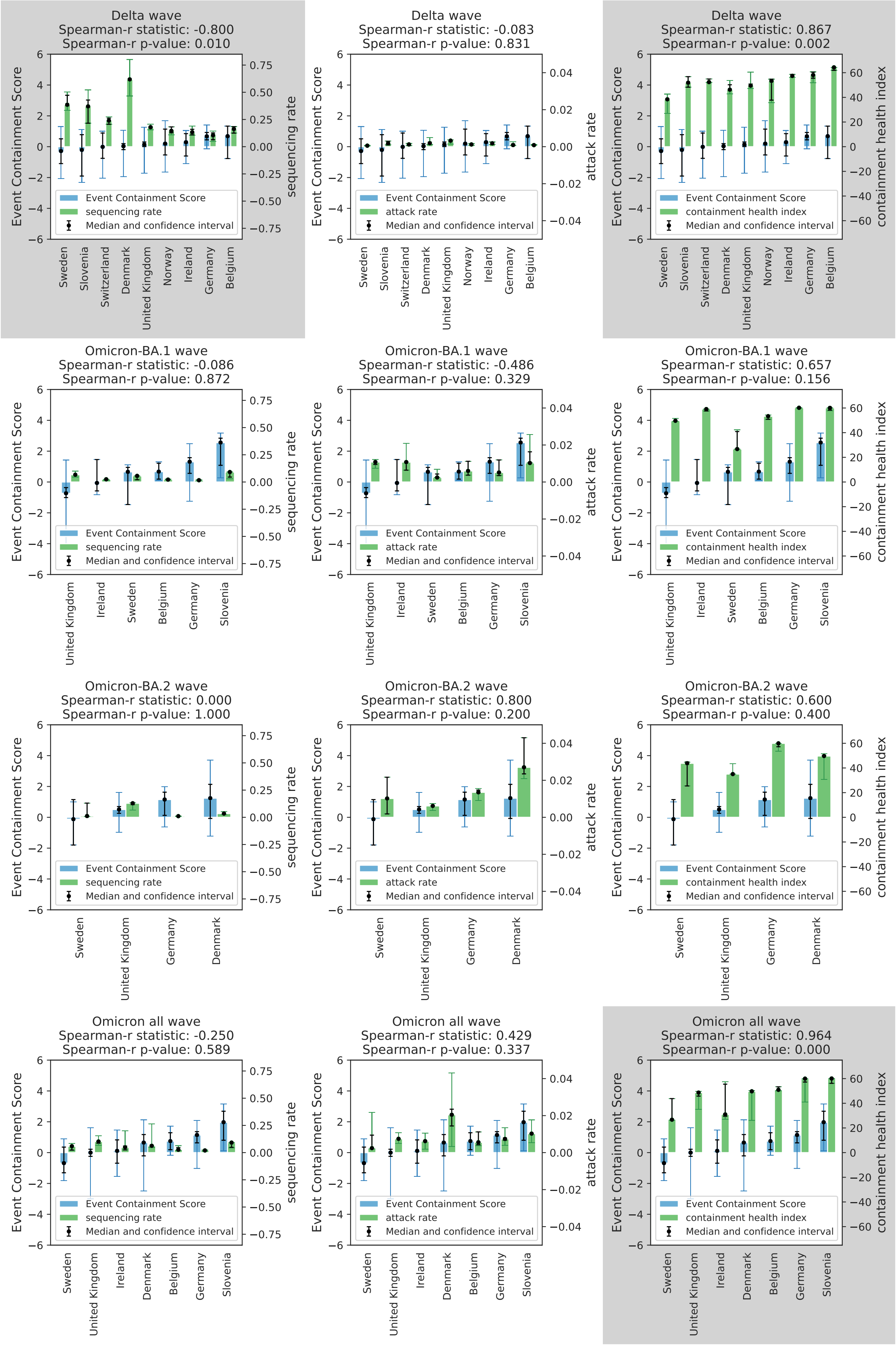

Figure B.5: The figure shows how Figure 4 of the main text would look like if $m = 2$ chosen instead of the default value ($m = 5$) when matching baseline events to superspreading events for the Delta and Omicron waves. Grey background signifies a statistically significant correlation between the MECS and the exogenous variable. Error bar definitions and all plotting parameters are the same as in Figure 4.

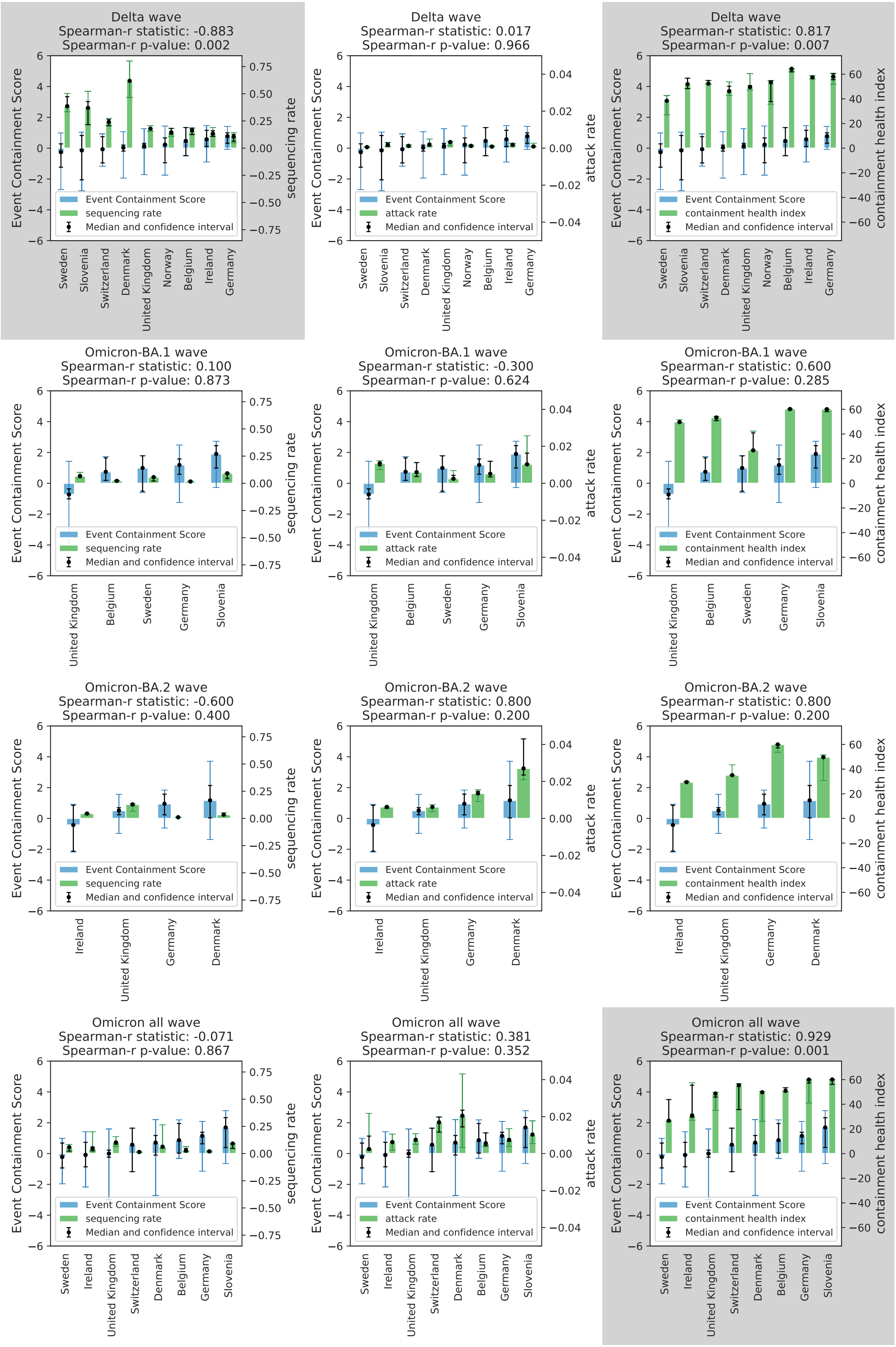

Figure B.6: The figure shows how Figure 4 of the main text would look like if $m = 10$ chosen instead of the default value ($m = 5$) when matching baseline events to superspreading events for the Delta and Omicron waves. Grey background signifies a statistically significant correlation between the MECS and the exogenous variable. Error bar definitions and all plotting parameters are the same as in Figure 4.

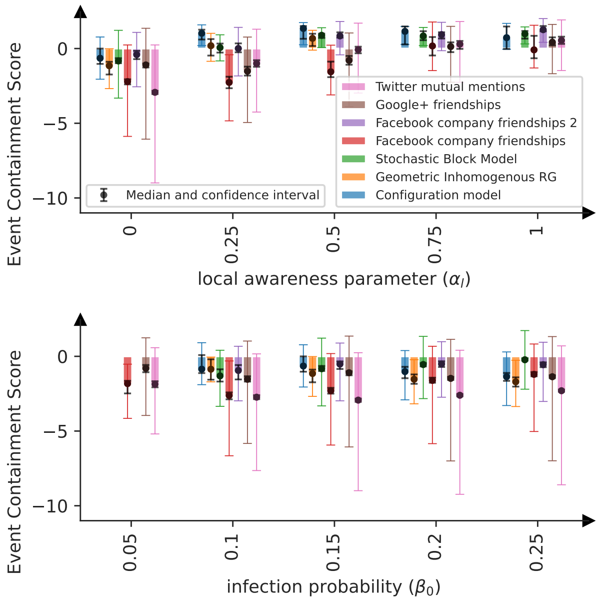

Figure B.7: ECS values computed on synthetically generated genetic sequence data similarly to Figure 3 of the main text, except with linear local and global awareness functions (see Methods for the precise function definition). The sample size, error bar definitions and all plotting parameters are the same as in Figure 3.

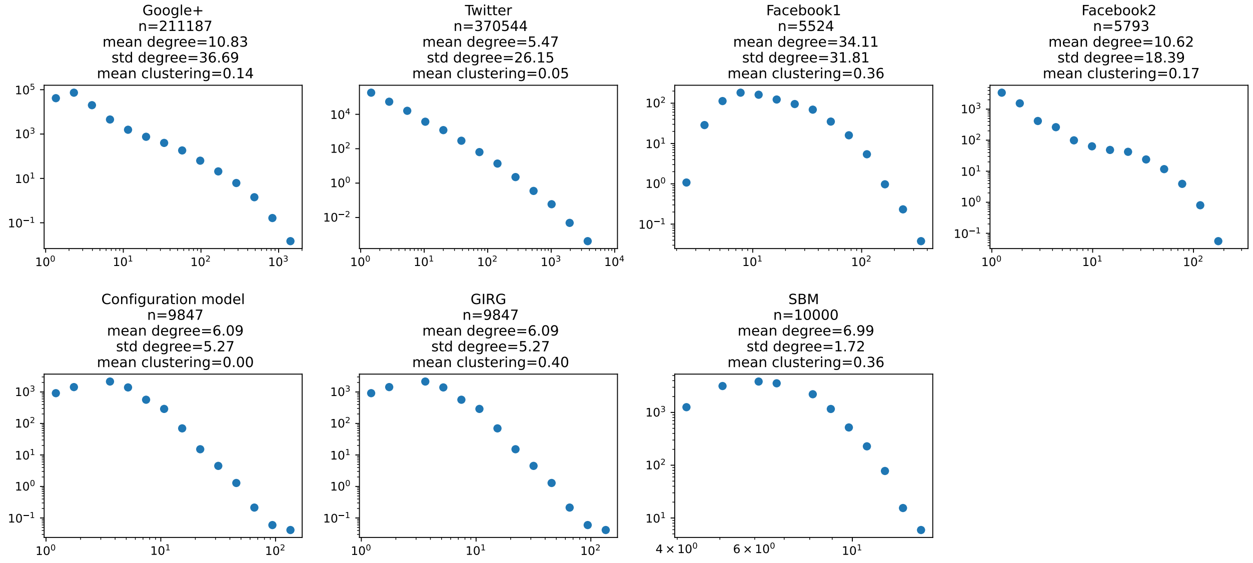

Figure B.8: Size, degree distribution and average clustering coefficient of the selected real and synthetic networks
