# Supplementary material for: Epidemic-induced local awareness behavior inferred from surveys and genetic sequence data
Source: Nat Commun. 2025 May 22;16:4758. doi: 10.1038/s41467-025-59508-5 (PMC12098690; doi:10.1038/s41467-025-59508-5)
Supplement: Supplementary file 2 — Description of Additional Supplementary Files [file 41467_2025_59508_MOESM2_ESM.pdf]

**Description of Additional Supplementary Files**

**Supplementary Data 1:** Source data for Figure 1a.

**Supplementary Data 2:** Source data for Figure 3.

**Supplementary Data 3:** An intermediate dataset containing the accession numbers of the sequences, the computed ECS values, and various additional metadata of the collision clusters corresponding to the detected superspreading events.

**Supplementary Data 4:** GISAID Supplemental Table containing further information on the genetic sequences used in this study.
