# Supplementary material for: Epidemic-induced local awareness behavior inferred from surveys and genetic sequence data
Source: Nat Commun. 2025 May 22;16:4758. doi: 10.1038/s41467-025-59508-5 (PMC12098690; doi:10.1038/s41467-025-59508-5)
Supplement: Supplementary file 7 — Reporting Summary [file 41467_2025_59508_MOESM7_ESM.pdf]

nature portfolio

Corresponding author(s): Gergely Odor

Last updated by author(s): Apr 14, 2025

Reporting Summary

Nature Portfolio wishes to improve the reproducibility of the work that we publish. This form provides structure for consistency and transparency in reporting. For further information on Nature Portfolio policies, see our Editorial Policies and the Editorial Policy Checklist.

Statistics

For all statistical analyses, confirm that the following items are present in the figure legend, table legend, main text, or Methods section.

n/a	Confirmed	
☐	☒	The exact sample size (*n*) for each experimental group/condition, given as a discrete number and unit of measurement
☒	☐	A statement on whether measurements were taken from distinct samples or whether the same sample was measured repeatedly
☐	☒	The statistical test(s) used AND whether they are one- or two-sided<br>*Only common tests should be described solely by name; describe more complex techniques in the Methods section.*
☐	☒	A description of all covariates tested
☐	☒	A description of any assumptions or corrections, such as tests of normality and adjustment for multiple comparisons
☐	☒	A full description of the statistical parameters including central tendency (e.g. means) or other basic estimates (e.g. regression coefficient) AND variation (e.g. standard deviation) or associated estimates of uncertainty (e.g. confidence intervals)
☐	☒	For null hypothesis testing, the test statistic (e.g. *F*, *t*, *r*) with confidence intervals, effect sizes, degrees of freedom and *P* value noted<br>*Give P values as exact values whenever suitable.*
☒	☐	For Bayesian analysis, information on the choice of priors and Markov chain Monte Carlo settings
☒	☐	For hierarchical and complex designs, identification of the appropriate level for tests and full reporting of outcomes
☐	☒	Estimates of effect sizes (e.g. Cohen's *d*, Pearson's *r*), indicating how they were calculated

Our web collection on statistics for biologists contains articles on many of the points above.

Software and code

Policy information about availability of computer code

Data collection	The survey data was collected via telephone, no software was used.
Data analysis	Our data analysis was performed in Python using standard data analysis and statistical packages. The code developed for this study is shared via Code Ocean (10.24433/CO.1189503.v1).

For manuscripts utilizing custom algorithms or software that are central to the research but not yet described in published literature, software must be made available to editors and reviewers. We strongly encourage code deposition in a community repository (e.g. GitHub). See the Nature Portfolio guidelines for submitting code & software for further information.

Data

Policy information about availability of data

All manuscripts must include a data availability statement. This statement should provide the following information, where applicable:

- Accession codes, unique identifiers, or web links for publicly available datasets
- A description of any restrictions on data availability
- For clinical datasets or third party data, please ensure that the statement adheres to our policy

All genome sequences and associated metadata are published in GISAID's EpiCoV database. To view the contributors of each individual sequence with details such as accession number, Virus name, Collection date, Originating Lab and Submitting Lab and the list of Authors, visit https://doi.org/10.55876/gis8.240404rn. Data on the Oxford Stringency Index are available at: https://github.com/OxCGRT/covid-policy-tracker/, and on the number of reported COVID-19 cases at https://

github.com/CSSEGISandData/COVID-19/tree/master. The MASZK survey data shown in Figure 1 is shared as supplementary data. The raw MASZK survey data cannot be shared openly due to privacy regulations but they may be available upon request at karsaim@ceu.edu, after the signature of non-disclosure agreements with the data owners.

Research involving human participants, their data, or biological material

Policy information about studies with human participants or human data. See also policy information about sex, gender (identity/presentation), and sexual orientation and race, ethnicity and racism.

Reporting on sex and gender	No information about sex or gender has been used in our analysis of the MASZK dataset or the GISAID dataset.
Reporting on race, ethnicity, or other socially relevant groupings	No information race, ethnicity, or other socially relevant groupings has been used in our analysis of the MASZK dataset or the GISAID dataset.
Population characteristics	See above
Recruitment	The MASZK data was collected via cross-sectional anonymous representative phone surveys using the CATI methodology and involved a 1000 large nationally representative sample each month. During the data collection, participants were not asked information that could be used for their re-identification.
Ethics oversight	The MASZK data collection was fully complying with the actual European and Hungarian privacy data regulations and was approved by the Hungarian National Authority for Data Protection and Freedom of Information [42 in main text], and also by the Health Science Council Scientific and Research Ethics Committee (resolution number IV/3073- 1 /2021/EKU).

Note that full information on the approval of the study protocol must also be provided in the manuscript.

Field-specific reporting

Please select the one below that is the best fit for your research. If you are not sure, read the appropriate sections before making your selection.

☐ Life sciences ☒ Behavioural & social sciences ☐ Ecological, evolutionary & environmental sciences

For a reference copy of the document with all sections, see nature.com/documents/nr-reporting-summary-flat.pdf

Behavioural & social sciences study design

All studies must disclose on these points even when the disclosure is negative.

Study description	We study local awareness behavior in two main datasets. As part of the MASZK telephone survey (ref. 17 in the main text) we asked about the self-reported awareness behavior of the participants directly. In the GISAID genetic sequence dataset (ref. 28), we inferred awareness behavior indirectly, based on patterns in the infection spread.
Research sample	Both datasets were intended to be representative samples of the general population at the national level. The MASZK dataset was confirmed to be representative for the Hungarian population aged 18 or older by gender, age, education and domicile. In the GISAID dataset, we focused on the countries where the sampling rate was the highest.
Sampling strategy	The sampling strategy of the MASZK dataset was controlled by the survey research company, and the sampling strategy of the GISAID dataset depended on the contributing research laboratory or institute that uploaded the samples.
Data collection	The MASZK data was collected via cross-sectional anonymous representative phone surveys using the CATI methodology and involved a 1000 large nationally representative sample each month. During the data collection, participants were not asked information that could be used for their re-identification.
Timing	Data was collected once per a month during one week for 9 months, namely: October 2021, November 2021, December 2021, January 2022, February 2022, March 2022, April 2022, May 2022, June 2022. We downloaded the entire GISAID dataset from January 2020 until March 2023.
Data exclusions	In the GISAID dataset, we excluded countries where the sampling rate was lower than 2%, and where we did not detect at least 20 superspreading events.
Non-participation	The survey response rate was 49 percent, which is expressly higher than the average response rate (being between 15-20 percent) of telephone surveys in Hungary.
Randomization	No randomization was performed.

Reporting for specific materials, systems and methods

We require information from authors about some types of materials, experimental systems and methods used in many studies. Here, indicate whether each material, system or method listed is relevant to your study. If you are not sure if a list item applies to your research, read the appropriate section before selecting a response.

Materials & experimental systems

n/a	Involved in the study
☒	☐ Antibodies
☒	☐ Eukaryotic cell lines
☒	☐ Palaeontology and archaeology
☒	☐ Animals and other organisms
☒	☐ Clinical data
☒	☐ Dual use research of concern
☒	☐ Plants

Methods

n/a	Involved in the study
☒	☐ ChIP-seq
☒	☐ Flow cytometry
☒	☐ MRI-based neuroimaging

Plants

Seed stocks	*Report on the source of all seed stocks or other plant material used. If applicable, state the seed stock centre and catalogue number. If plant specimens were collected from the field, describe the collection location, date and sampling procedures.*
Novel plant genotypes	*Describe the methods by which all novel plant genotypes were produced. This includes those generated by transgenic approaches, gene editing, chemical/radiation-based mutagenesis and hybridization. For transgenic lines, describe the transformation method, the number of independent lines analyzed and the generation upon which experiments were performed. For gene-edited lines, describe the editor used, the endogenous sequence targeted for editing, the targeting guide RNA sequence (if applicable) and how the editor was applied.*
Authentication	*Describe any authentication procedures for each seed stock used or novel genotype generated. Describe any experiments used to assess the effect of a mutation and, where applicable, how potential secondary effects (e.g. second site T-DNA insertions, mosiacism, off-target gene editing) were examined.*
